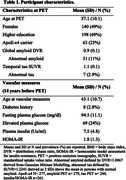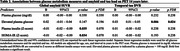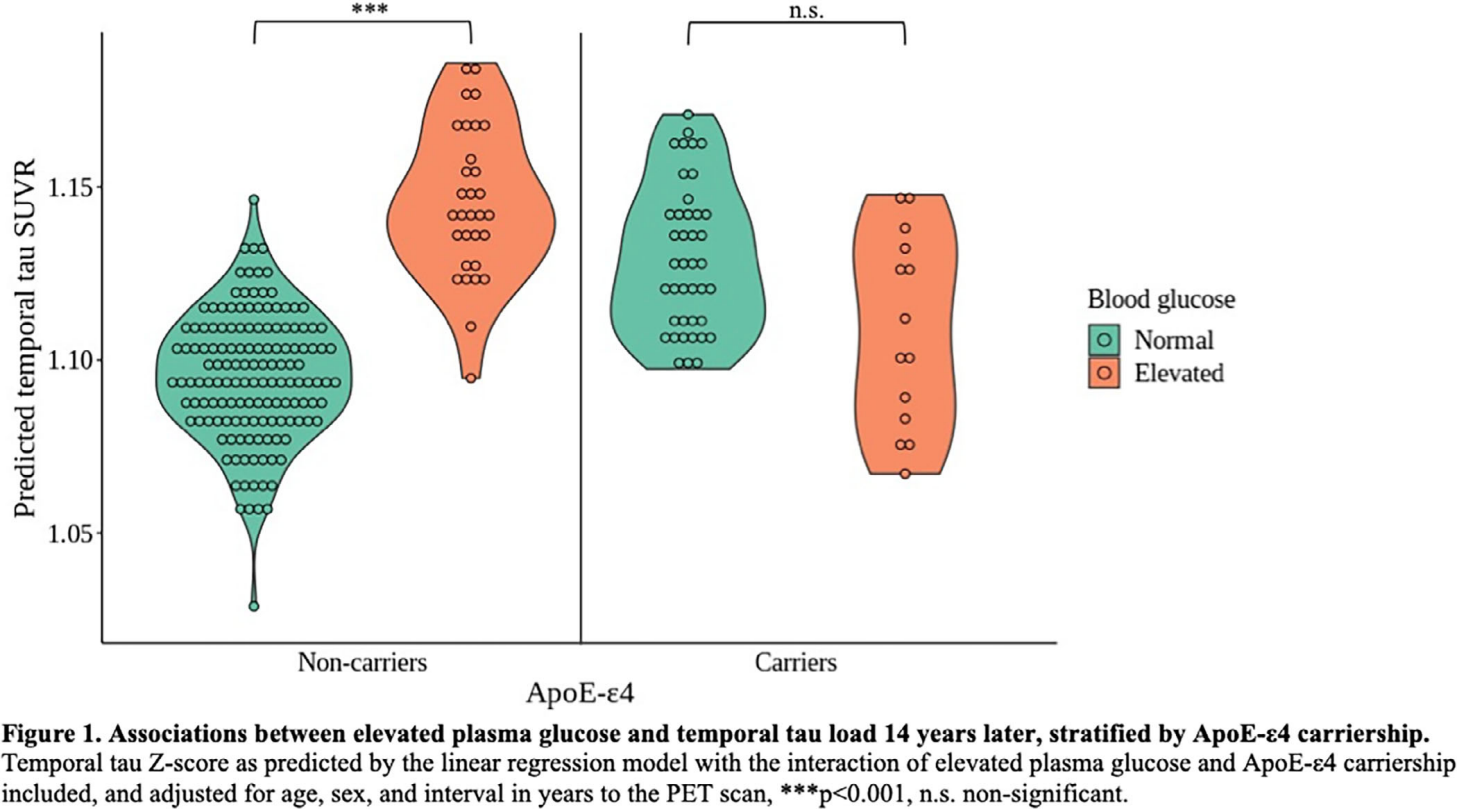# Associations between glucose metabolism measures and amyloid and tau load on PET 14 years later: Findings from the Framingham Heart Study

**DOI:** 10.1002/alz.090650

**Published:** 2025-01-09

**Authors:** Veerle van Gils, Qiushan Tao, Ting Fang Alvin Ang, Christina B. Young, Elizabeth Mormino, Wei Qiao Qiu, Pieter Jelle Visser, Rhoda Au, Willemijn J. Jansen, Stephanie J. B. Vos

**Affiliations:** ^1^ Alzheimer Center Limburg, School for Mental Health and Neuroscience, Maastricht University, Maastricht Netherlands; ^2^ Department of Pharmacology, Physiology & Biophysics, Boston University Chobanian & Avedisian School of Medicine, Boston, MA USA; ^3^ Slone Epidemiology Center, Boston University Chobanian & Avedisian School of Medicine, Boston, MA USA; ^4^ Framingham Heart Study, Boston University Chobanian & Avedisian School of Medicine, Boston, MA USA; ^5^ Department of Anatomy & Neurobiology, Boston University Chobanian & Avedisian School of Medicine, Boston, MA USA; ^6^ Stanford University School of Medicine, Stanford, CA USA; ^7^ Department of Neurology and Neurological Sciences, Stanford University School of Medicine, Stanford, CA USA; ^8^ Alzheimer Center and Department of Neurology, Amsterdam Neuroscience Campus, VU University Medical Center, Amsterdam Netherlands; ^9^ Department of Epidemiology, Boston University School of Public Health, Boston, MA USA; ^10^ Department of Neurology, Boston University Chobanian & Avedisian School of Medicine, Boston, MA USA

## Abstract

**Background:**

Type 2 diabetes and glucose metabolism have previously been linked to cognitive decline and higher risk of developing Alzheimer’s disease (AD) dementia. Yet, the relation of glucose metabolism with amyloid and tau pathology remains unclear. This knowledge will help understanding the importance of glucose regulation in relation to AD. Therefore, we aimed to investigate whether earlier age glucose metabolism measures are associated with later age amyloid and tau measures on PET.

**Method:**

We included 288 participants (mean age= 43.1, SD=10.7, range 20‐70 years) without dementia from the Framingham Heart Study (FHS), who had data available on glucose metabolism measures, i.e. continuous plasma glucose, elevated plasma glucose (>100mg/dl), plasma insulin, and homeostatic model assessment for insulin resistance (HOMA‐IR), and a PET measure of global amyloid and/or temporal tau 14 years later (range 11‐17 years). We performed linear regression analyses to test associations of each glucose metabolism measure with amyloid or tau uptake on PET, adjusted for age, sex, and exact years of time interval. For significant findings, we explored whether age, sex, ApoE‐ε4 allele carriership or amyloid load modified the associations.

**Result:**

Demographics are shown in Table 1. Our findings indicated that elevated plasma glucose was associated with greater tau load in the brain 14 years later (B [95%CI] = 0.03 [0.01 – 0.05], p = 0.006). Findings were independent from age, sex, and amyloid load. The association was only observed in ApoE‐ε4 non‐carriers (B [95%CI] = ‐0.08 [‐0.12 – ‐0.03], p=0.001, Figure 1). Higher plasma insulin and HOMA‐IR were associated with decreased amyloid load after 14 years (B [95%CI] = ‐0.01 [‐0.02 – ‐0.00], p = 0.035 and B [95%CI] = ‐0.01 [‐0.02 – ‐0.00], p = 0.034, respectively), but this association attenuated after false discovery rate (FDR) correction (both p=0.07) and adjustment for ApoE‐ε4 carriership (insulin p=0.057 and HOMA‐IR p=0.053). No other associations were found (Table 2).

**Conclusion:**

Our findings suggest that impaired glucose metabolism is associated with increased tau pathology later in life, independent from amyloid pathways. These findings suggest that glucose metabolism regulation is important to prevent neuropathology later in life.